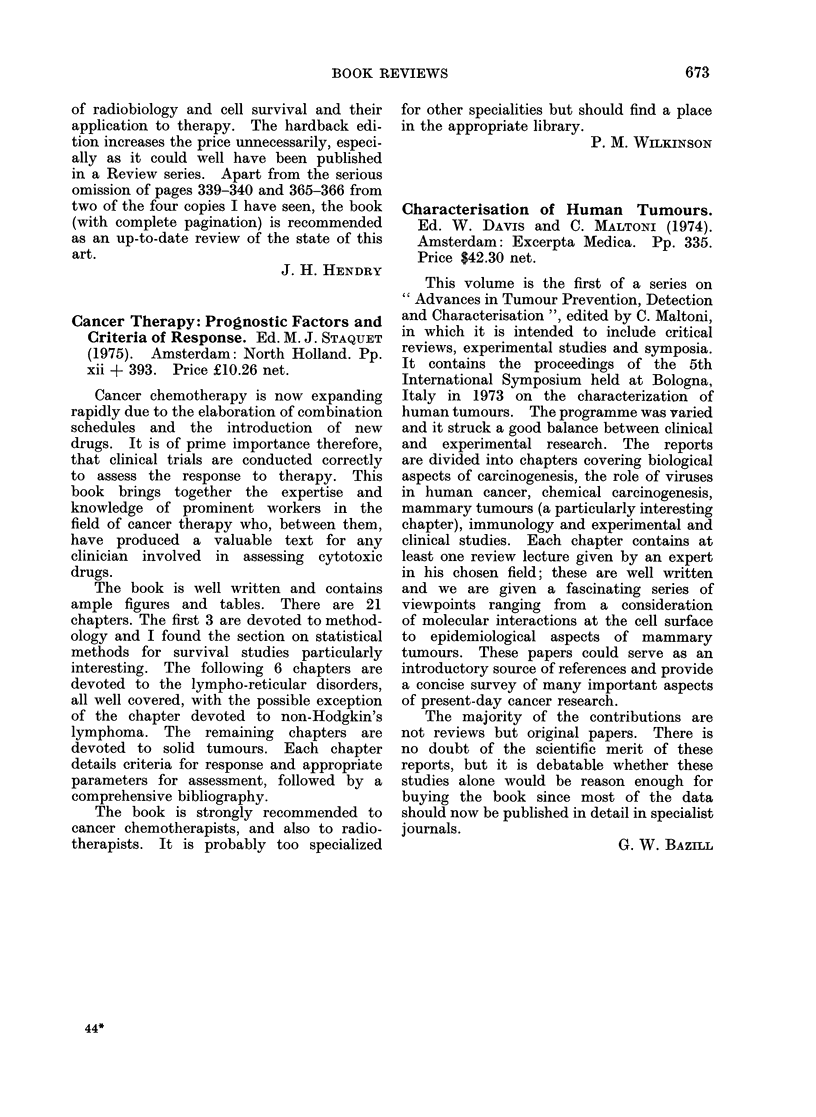# Cancer Therapy: Prognostic Factors and Criteria of Response

**Published:** 1976-06

**Authors:** P. M. Wilkinson


**Cancer Therapy: Prognostic Factors and Criteria of Response.** Ed. M. J. STAQUET (1975). Amsterdam: North Holland. Pp. xii + 393. Price £10.26 net.

Cancer chemotherapy is now expanding rapidly due to the elaboration of combination schedules and the introduction of new drugs. It is of prime importance therefore, that clinical trials are conducted correctly to assess the response to therapy. This book brings together the expertise and knowledge of prominent workers in the field of cancer therapy who, between them, have produced a valuable text for any clinician involved in assessing cytotoxic drugs.

The book is well written and contains ample figures and tables. There are 21 chapters. The first 3 are devoted to methodology and I found the section on statistical methods for survival studies particularly interesting. The following 6 chapters are devoted to the lympho-reticular disorders, all well covered, with the possible exception of the chapter devoted to non-Hodgkin's lymphoma. The remaining chapters are devoted to solid tumours. Each chapter details criteria for response and appropriate parameters for assessment, followed by a comprehensive bibliography.

The book is strongly recommended to cancer chemotherapists, and also to radiotherapists. It is probably too specialized for other specialities but should find a place in the appropriate library.

P. M. WILKINSON